# Massively parallel sequencing identifies a previously unrecognized X-linked disorder resulting in lethality in male infants owing to amino-terminal acetyltransferase deficiency

**DOI:** 10.1186/gb-2011-12-s1-p13

**Published:** 2011-09-19

**Authors:** Alan F Rope, Kai Wang, Rune Evjenth, Jinchuan Xing, Jennifer J Johnston, Jeffrey J Swensen, W Evan Johnson, Barry Moore, Chad D Huff, Lynne M Bird, John C Carey, John M Opitz, Cathy A Stevens, Christa Schank, Heidi Deborah Fain, Reid Robison, Brian Dalley, Steven Chin, Sarah T South, Theodore J Pysher, Lynn B Jorde, Hakon Hakonarson, Johan R Lillehaug, Leslie G Biesecker, Mark Yandell, Thomas Arnesen, Gholson J Lyon

**Affiliations:** 1 Department of Pediatrics (Medical Genetics), University of Utah School of Medicine, Salt Lake City, UT, USA; 2 Center for Applied Genomics, Children’s Hospital of Philadelphia, Philadelphia, PA , USA; 3 Present address: Zilkha Neurogenetic Institute, Department of Psychiatry and Preventive Medicine, University of Southern California, Los Angeles, CA, USA; 4 Department of Molecular Biology, University of Bergen, N-5020 Bergen, Norway; 5 Eccles Institute of Human Genetics, University of Utah, Salt Lake City, UT, USA; 6 Genetic Disease Research Branch, National Human Genome Research Institute, National Institutes of Health, Bethesda, MD, USA; 7 Department of Pathology, University of Utah, Salt Lake City, UT, USA; 8 ARUP Laboratories, Salt Lake City, Utah, USA; 9 Department of Statistics, Brigham Young University, Provo, Utah, USA; 10 Rady Children’s Hospital and University of California, San Diego, Department of Pediatrics, San Diego, CA, USA; 11 Department of Obstetrics and Gynecology, University of Utah, Salt Lake City, UT, USA; 12 Department of Pediatrics, University of Tennessee College of Medicine, Chattanooga, TN, USA; 13 Department of Psychiatry, University of Utah, Salt Lake City, UT, USA; 14 Huntsman Cancer Institute, Salt Lake City, UT, USA; 15 Department of Surgery, Haukeland University Hospital, N-5021 Bergen, Norway; 16 New York University Child Study Center, New York, NY, USA; 17 Present address: Center for Applied Genomics, Children’s Hospital of Philadelphia, Philadelphia, PA , USA

## Background

Individuals II-1 and II-6 from family 1 presented in the mid-1980s to the University of Utah Medical Center. These boys had a striking similarity to each other, with a range of shared clinical manifestations, but the disease they presented with was not a recognized syndrome. Both boys subsequently died in infancy. X-linked inheritance was confirmed in the next generation, when individuals III-4 and III-7 presented with the same disease. Their aged appearance was the most striking part of the disease.

## Methods

We describe two parallel genetic research efforts that converged on the same gene variant. Exon capture was carried out on samples from two families, using a commercially available in-solution method (Agilent’s SureSelect Human X Chromosome kit) as per the manufacturer’s guidelines with minor modifications to generate sequencing libraries (Illumina). We also used a recently developed tool, the Variant Annotation, Analysis and Selection Tool (VAAST), which identifies disease-causing variants, to analyze the exon capture data from family 1. Our analysis applied a disease model that did not require complete penetrance or locus homogeneity. We restricted the expected allele frequency of putative disease-causing variants within the control genomes to 0.1% or lower. The background file used in the analysis is composed of variants from dbSNP (version 130), 189 genomes from the 1000 Genomes Project, the 10Gen Data Set, 184 Danish exomes and 40 whole genomes from the Complete Genomics Diversity Panel. VAAST candidate gene prioritization analysis was performed using the likelihood ratio test under the dominant inheritance model, assuming an expected allele frequency of 0.1% or lower for the causal variant in the general population. After masking out loci of potentially low variant quality, single nucleotide variations in each gene were scored as a group. The significance level was assessed using individual permutation tests.

## Results

We identified a family with a previously undescribed lethal X-linked disorder of infancy comprising a distinct combination of an aged appearance, craniofacial anomalies, hypotonia, global developmental delays, cryptorchidism, cardiac arrhythmia and cardiomyopathy. We used X-chromosome exon sequencing and a recently developed probabilistic disease-gene discovery algorithm to identify a missense variant in *NAA10*, which encodes the catalytic subunit of the major human amino-terminal acetyltransferase (NAT; also known as hNaa10p). More recently, we became aware that a parallel effort on a second unrelated family converged on the same variant. The absence of this variant in controls, the amino acid conservation of this region of the protein, the predicted disruptive change and the co-occurrence in two unrelated families with the same rare disorder suggest that this is the pathogenic mutation. We confirmed this by demonstrating that the mutant hNaa10p had significantly impaired biochemical activity, and we therefore conclude that a reduction in acetylation by hNaa10p causes this disease.

## Conclusions

This is one of the first uses of next-generation sequencing to identify the genetic basis of a previously unrecognized X-linked syndrome. It is also the first evidence of a human genetic disorder resulting from direct impairment of amino-terminal acetylation, one of the most common protein modifications in humans. We have also demonstrated that a probabilistic disease-gene discovery algorithm (VAAST) can readily identify and characterize the genetic basis of this syndrome.

